# Inpatient consultations with the vascular and endovascular surgery team at an academic tertiary hospital

**DOI:** 10.1590/1677-5449.20210159

**Published:** 2022-05-06

**Authors:** Daniel Urban Raymundo, Marcelo Bellini Dalio, Mauricio Serra Ribeiro, Edwaldo Edner Joviliano

**Affiliations:** 1 Universidade de São Paulo – USP, Faculdade de Medicina de Ribeirão Preto, Departamento de Cirurgia e Anatomia, Divisão de Cirurgia Vascular e Endovascular, Ribeirão Preto, SP, Brasil.

**Keywords:** inpatients, consultations, vascular, endovascular, surgery, tertiary, hospital, care, healthcare, pacientes internados, consultas, vascular, endovascular, cirurgia, terciário, hospital, atendimento, serviços de saúde

## Abstract

**Background:**

Inpatient consultations are a fundamental component of practice in tertiary care centers. However, such consultations demand resources, generating a significant workload.

**Objectives:**

To investigate the profile of inpatient consultations requested by other specialties and provided by the Vascular and Endovascular Surgery team at an academic tertiary hospital.

**Methods:**

Prospective observational study.

**Results:**

From May 2017 to May 2018, 223 consultations were provided, representing 2.2% of the workload. Most consultations were requested by Oncology (16.6%), Hematology (9.9%), Nephrology (9.0%), and Cardiology (6.3%). The leading reasons for inpatient consultation were: need for vascular access (51.1%) and requests to evaluate a vascular disease (48.9%). Acute venous diseases accounted for 19.3% of consultations, chronic arterial diseases for 14.8%, acute arterial diseases for 7.2%, diabetic feet for 5.4%, and chronic venous diseases accounted for 2.2%. Surgical treatment was performed in 57.0%, either conventional (43.9%) or endovascular (13.0%). Almost all (98.2%) patients’ issues were resolved.

**Conclusions:**

Inpatient consultations with the Vascular and Endovascular Surgery team in a tertiary academic hospital accounted for 2.2% of the team’s entire workload. Most patients were elective and underwent low-complexity elective surgical procedures. There may be an opportunity to improve healthcare, redirecting these patients to the outpatient flow.

## RESUMO

**Introdução:**

Interconsultas são um componente fundamental da prática clínica em centros de atendimento terciários. No entanto, esse tipo de consulta requer recursos, resultando em uma alta carga de trabalho.

**Objetivo:**

Investigar o perfil das interconsultas solicitadas por outros departamentos e realizadas pela equipe de Cirurgia Vascular e Endovascular em um hospital universitário terciário.

**Métodos:**

Estudo observacional prospectivo.

**Resultados:**

De maio de 2017 a maio de 2018, foram realizadas 223 consultas, correspondendo a 2,2% da carga de trabalho. A maioria das consultas foram solicitadas pelos departamentos de Oncologia (16,6%), Hematologia (9,9%), Nefrologia (9,0%) e Cardiologia (6,3%). As principais razões das interconsultas foram a necessidade de acesso vascular (51,1%) e de avaliação de doenças vasculares (48,9%). As doenças venosas agudas corresponderam a 19,3% das avaliações; as doenças arteriais crônicas, a 14,8%; as doenças arteriais agudas, a 7,2%; o pé diabético, a 5,4%; e as doenças venosas crônicas corresponderam a 2,2%. Foi realizado tratamento cirúrgico em 57,0% dos casos, tanto convencional (43,9%) quanto endovascular (13,0%). Foram resolvidos os problemas de quase todos os pacientes (98,2%).

**Conclusão:**

As interconsultas realizadas pela equipe de Cirurgia Vascular e Endovascular em um hospital universitário terciário corresponderam a 2,2% da carga de trabalho total. A maioria dos pacientes eram eletivos e foram submetidos a procedimentos cirúrgicos eletivos de baixa complexidade. O redirecionamento desses pacientes para o atendimento ambulatorial poderia auxiliar na melhoria dos serviços de saúde.

## INTRODUCTION

Inpatient consultations are a fundamental component of clinical practice in tertiary care centers. In these large hospitals, patients generally have multiple comorbidities and require multidisciplinary care. Inpatient consultations allow doctors to seek experienced colleagues from other specialties to manage complex cases. They also encourage discussion and learning, which is crucial in academic hospitals, where junior doctors are undergoing training.^1^ As the population ages, cardiovascular diseases become more prevalent.^2^ Vascular and Endovascular Surgery teams frequently provide inpatient consultations as a part of their practice.^3^

However, such consultations demand time and human resources, constituting a significant workload over and above the usual day-to-day duties of individual teams. This is a markedly overlooked aspect of service provision, as available data related to this activity are scarce.^4^

Understanding these inpatient consultation profiles is essential to organizing human resources, equipment, and related supplies. It is also crucial to identify possible failures and effect improvements in the system. In a specialty with an emerging demand and growing costs such as Vascular and Endovascular Surgery, this knowledge is hugely relevant.^5^ This study aimed to investigate the profile of inpatient consultations requested by other specialties and provided by the Vascular and Endovascular Surgery team at an academic tertiary hospital. We also intended to identify items needed to promote improvements in healthcare.

## METHODS

### Study design

We conducted a prospective observational study of inpatient consultations with the Vascular and Endovascular Surgery team at our institution. The institutional Ethics and Research Committee approved the study (number 1.996.150), which is in accordance with the Helsinki Declaration and with local ethical guidelines. Individual informed consent was waived by the committee.

### Patients

All patients who had inpatient consultations with the Vascular and Endovascular Surgery team requested by other specialties from May 2017 to May 2018 were included.

### Structure of the Vascular and Endovascular Surgery division

Our institution is a 500-bed public tertiary care hospital, a regional referral center for more than 30 specialties. It is focused on elective hospital admissions, based on referrals from the public health system. There is no on-site emergency department and therefore the hospital does not receive acute trauma or other medical or surgical emergencies. It is also an academic hospital, where medical students, residents, and post-graduate doctors are trained. The Vascular and Endovascular Surgery team comprises residents and staff and is available for inpatient consultations 24 hours a day.

### Data collected and definitions

We collected demographic data, comorbidities, source specialty, the reason for the consultation, and the vascular disease diagnosed for each patient included. We also determined whether the consultation was elective, urgent, or emergent. The treatment adopted and the patient’s progress were also noted.

The source specialty was considered to be the team that first admitted the patient. It is important to note this information, since tertiary care patients generally have multiple diseases and require a multidisciplinary approach. The reason for the consultation was obtained from the written consultation request. The vascular disease diagnosed, when present, was the disease determined by the vascular team after the consultation had taken place. The vascular diseases diagnosed were classified as acute arterial disease, chronic arterial disease, acute venous disease, chronic venous disease, or diabetic foot.

A consultation was considered elective if the request was fulfilled in more than 24 hours. Urgent consultations were those provided in less than 24 hours. Emergent consultations were those required immediately. The treatment adopted was classified as surgical or medical. Surgical procedures were subdivided into conventional or endovascular. Outcomes were classified in three categories: 1) Resolution, if the treatment adopted resulted in improvement or resolution of the issue; 2) Clinical deterioration, if the patient underwent vascular-related clinical deterioration or if the issue prompting the request was not resolved; and 3) Death while in hospital.

### Statistical analysis

Data were expressed as absolute numbers and proportions. The 95% confidence interval was calculated using the Wald method.

## RESULTS

**Figure 1:**
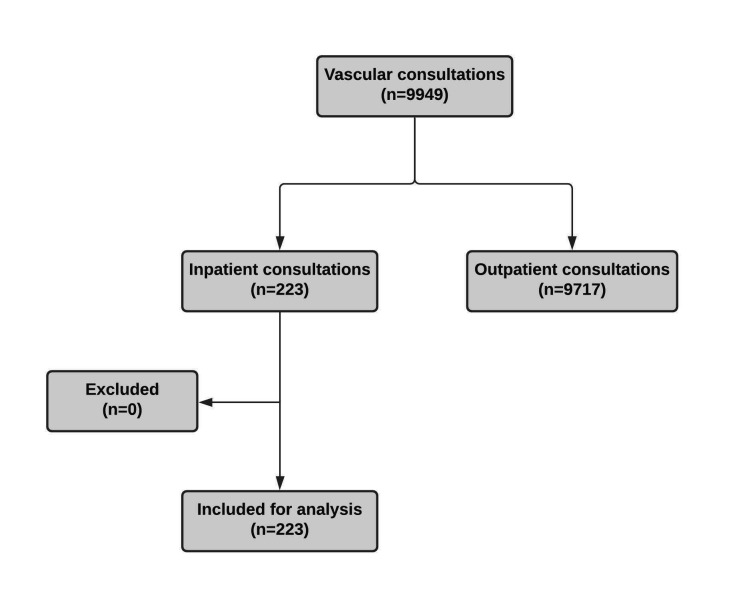
Study flowchart.

During the study period, 223 inpatient consultations were requested and provided by the vascular team. In the same period, the same team provided 9717 consultations on an outpatient basis, making a total of 9940 consultations. Inpatient consultations accounted for 2.2% of the team’s entire workload. No patients were excluded from the analysis. Figure 1 describes the study. The average age of individuals was 50.7 (± 19.4) years, and 50.5% were female. Cancer was the most frequent comorbidity, in 83 individuals (37.2%). Systemic arterial hypertension was the second most prevalent condition, present in 73 individuals (32.7%). Diabetes mellitus was the third most frequent condition, present in 44 patients (19.7%). Heart failure (12.1%), smoking (8.6%), dyslipidemia (7.6%), and obesity (2.5%) were other frequently observed comorbidities.

Table 1 presents the collected data. Most consultations were requested by medical specialties (79.4%). Consultations from Oncology (16.6%), Hematology (9.9%), Nephrology (9.0%), and Cardiology (6.3%) were the most common. Surgical specialties requested 46 consultations (20.6%). General surgery (4.0%), Obstetrics & Gynecology (3.6%), and Orthopedics (3.6%) were the surgical teams that called the vascular team more commonly. There were no intraoperative consultations. Elective consultations accounted for almost three quarters of the total (72.6%). A quarter of consultations were urgent and only 5 (2.2%) were emergencies.

**Table 1:** Inpatient consultations with the Vascular and Endovascular Surgery team at an academic tertiary hospital over a 1 year period. Source specialty, urgency, reason for consultation, treatment, and outcome of the patients seen.

**Source specialty**	**n=223**	**95% CI** *
Oncology	37 (16.6%)	12.25-22.06
Hematology	22 (9.9%)	6.55-14.54
Nephrology	20 (9.0%)	5.82-13.51
Cardiology	14 (6.3%)	3.69-10.34
Nutrition and Dietetics	10 (4.5%)	2.35-8.16
Infectious Diseases	9 (4.0%)	2.03-7.60
General Surgery	9 (4.0%)	2.03-7.60
Endocrinology	8 (3.6%)	1.71-7.03
Obstetrics & Gynecology	8 (3.6%)	1.71-7.03
Orthopedics	8 (3.6%)	1.71-7.03
Adult Intensive Care	7(3.1%)	1.40-6.46
Dermatology	6 (2.7%)	1.10-5.88
Immunology	6 (2.7%)	1.10-5.88
Proctology	6 (2.7%)	1.10-5.88
Internal Medicine	5 (2.2%)	0.81-5.29
Neurology	5 (2.2%)	0.81-5.29
Cardiac surgery	4 (1.8%)	0.54-4.68
Urology	4 (1.8%)	0.54-4.68
Gastroenterology	3 (1.3%)	0.27-4.06
Pediatrics	3 (1.3%)	0.27-4.06
Pneumology	3 (1.3%)	0.27-4.06
Mastology	3 (1.3%)	0.27-4.06
Geriatrics	2 (0.9%)	0.03-3.42
Neonatology	2 (0.9%)	0.03-3.42
Pediatric Intensive Care	2 (0.9%)	0.03-3.42
Head and Neck Surgery	2 (0.9%)	0.03-3.42
Interventional Radiology	2 (0.9%)	0.03-3.42
Other	13 (5.8%)	3.35-9.80
		
**Urgency**		
Elective	162 (72.6%)	66.44-78.09
Urgent	56 (25.1%)	19.86-31.21
Emergent	5 (2.2%)	0.81-5.29
		
**Reason for the consultation**		
Need for vascular access	114 (51.1%)	44.60-57.61
Elective chemotherapy long-term catheter	83 (37.2%)	31.14-43.73
Elective vascular access for hemodialysis	18 (8.1%)	5.10-12.46
Urgent vascular access	13 (5.8%)	3.35-9.80
Vascular diseases	109 (48.9%)	42.39-55.40
Acute venous diseases	43 (19.3%)	14.62-24.99
Chronic arterial diseases	33 (14.8%)	10.70-20.09
Acute arterial diseases	16 (7.2%)	4.39-11.41
Acute limb ischemia	14 (6.3%)	3.69-10.34
Ruptured aortic aneurysms	2 (0.9%)	0.03-3.42
Diabetic foot	12 (5.4%)	3.01-9.26
Chronic venous diseases	5 (2.2%)	0.81-5.29
		
**Treatment**		
Surgical	127 (57%)	50.39-63.28
Conventional	98 (43.9%)	37.59-50.51
Endovascular	29 (13.0%)	9.17-18.10
Medical	96 (43.0%)	36.72-49.61
		
**Outcome**		
Resolution	219 (98.2%)	95.32-99.46
Clinical deterioration	1 (0.4%)	0.01-2.75
Death	3 (1.3%)	0.27-4.06

* 95% CI = 95% confidence interval, obtained by the Wald method.

The leading reason for inpatient consultation was a need for vascular access (51.1%). Three situations were observed: Oncology patients needing elective long-term catheters for chemotherapy (37.2%), chronic kidney disease patients needing elective vascular access for hemodialysis (8.1%), and patients who needed urgent vascular access (5.8%).

A request to evaluate a vascular disease was the second most common reason for consultation (48.9%). Acute venous disease, more specifically, deep venous thrombosis was the most frequent condition (19.3%). Acute deep venous thrombosis usually affected patients in the postoperative period, with cancer, or both. Patients with critical limb ischemia were classified as chronic arterial diseases (14.8%). Acute arterial diseases (7.2%) were one of two different problems: acute limb ischemia or ruptured abdominal aortic aneurysms. Diabetic foot accounted for 5.4%. Varicose veins and ulcers were considered chronic venous diseases (2.2%).

After consultations, the vascular team performed surgery in 57.0% of the cases, either conventional (43.9%) or endovascular (13.0%). The remaining 43% of patients received medical treatment. Almost all (98.2%) patients’ issues were resolved. One patient presented clinical deterioration (0.4%). Three critical patients died during the hospital stay (1.3%).

## DISCUSSION

The inpatient consultations in our study could be classified into two groups: patients needing vascular access and patients presenting with a peripheral vascular disease. The patients in the first group usually came from Oncology or Hematology, had cancer, and required long-term catheters for chemotherapy. Also in this group, a small number of patients from Nephrology needed vascular access for hemodialysis. These patients were all elective, and all underwent an elective surgical procedure scheduled after the consultation. A few patients from the vascular access group required urgent access. Most of these patients came from the Intensive Care Unit and were typically critical. All had difficult venous access, which required specialist care. These constituted real urgent cases.

A high prevalence of vascular access inpatient consultations is expected in tertiary care hospitals, focused on elective treatments.^6^ These centers receive many patients with cancer and chronic kidney failure and procedures to obtain vascular access for chemotherapy and hemodialysis are in high demand. A similar analysis in a tertiary hospital also reported vascular access as the main reason for inpatient consultations.^7^

Patients who had a vascular disease that needed medical or surgical care formed a markedly heterogeneous group, coming from different specialties. The degree of urgency of the consultation varied and a significant number of patients were managed with medical treatment. A high prevalence of deep venous thrombosis and chronic arterial diseases was expected in a tertiary hospital such as ours. However, other authors did not share this finding. They reported trauma and acute arterial diseases as leading reasons for inpatient consultations.^4^^,^^7^ It should be emphasized that our hospital does not have an emergency department. Thus, trauma patients were not present in our study. Arterial emergencies were less frequent than in the above-cited studies.

More than half of the patients in this study required surgical treatment, either on an elective or on an urgent basis. This finding is expected for a surgical subspecialty such as Vascular and Endovascular Surgery. Similar findings were reported by others.^4^^,^^7^ Vascular inpatient consultations were highly effective, since most issues were resolved. Interestingly, there were no intraoperative vascular consultations, as have been reported by others.^8^

Inpatient consultations are time-consuming and can generate a significant workload for specialist teams in tertiary care hospitals.^9^ While the elective and emergency activities are readily demonstrated by admission and discharge data, theater activity, and outpatient caseloads, the workload generated by inpatient consultations is usually not captured by healthcare indicators, representing a “hidden workload”.^10^ In our study, inpatient consultations were only 2.2% of all consultations. In relative numbers, this seems to be very small. However, if we consider the time taken to see the patients, the time waiting for imaging exams, the time in surgery, the follow-up hospital visits time, and the time spent on outpatient follow-up consultations for all 233 patients, we can estimate a significant additional workload.

Analyzing inpatient consultations is crucial to quantify the “hidden workload” and to determine reference standards. Healthcare can be improved by optimizing the workload, particularly in environments where work time is monitored for service quality. Determining where the hospital service is inefficient can improve care quality, reducing needless requests for consultations. Unnecessary consultations can affect the Vascular and Endovascular Surgery service’s performance and coverage. In an academic environment, these unnecessary consultations can negatively influence the educational process and training efficiency.^11^^,^^12^

Most of our 223 inpatient consultations were elective, with no need to be provided urgently. As discussed above, patients needing chemotherapy catheters and hemodialysis access were elective and underwent low-complexity elective surgical procedures. Together, these patients represented 45.3% of all consultations. These patients could have been seen on an outpatient basis without requiring time from the team to carry out the assessment, thus causing the professional to move from another activity, which could directly or indirectly affect service logistics.^9^

These observations reveal an opportunity to improve inpatient consultation workflow and plan better use of human resources. One possible solution would be implementing a new outpatient flow for patients requiring vascular accesses on an elective basis. Indeed, this new flow would work according to the institution’s current referral guidelines. It would be simply a change in the team’s workflow and an increase in the number of surgeons available would not be necessary. Thus, requests for chemotherapy catheters and hemodialysis access would no longer be routine for inpatient consultation. With this elementary solution, we expect about a 40% decrease in consultation requests.

The main limitation of this study is that these findings reflect the situation at a tertiary academic hospital. These data may not represent the reality at other services, particularly private and non-academic hospitals.^11^ For some consultations, a single diagnosis was difficult to obtain because of multiple clinical conditions. This may have led to categorization errors. However, these limitations do not overshadow our findings. Our study was prospective and describes the reality at many centers around the world.

## CONCLUSION

Inpatient consultations provided by the Vascular and Endovascular Surgery team at an academic tertiary academic hospital equated to 2.2% of the team’s entire workload. Patients needing vascular access were the main reason for consultation. Most of these patients were elective and underwent low-complexity elective surgical procedures. There may be an opportunity to improve healthcare, redirecting these patients to the outpatient flow.
